# Development, feasibility and acceptability of a gamified cognitive DEvelopmental assessment on an E-Platform (DEEP) in rural Indian pre-schoolers – a pilot study

**DOI:** 10.1080/16549716.2018.1548005

**Published:** 2019-01-11

**Authors:** Supriya Bhavnani, Debarati Mukherjee, Jayashree Dasgupta, Deepali Verma, Dhanya Parameshwaran, Gauri Divan, Kamal Kant Sharma, Tara Thiagarajan, Vikram Patel

**Affiliations:** a Centre for Chronic Conditions and Injuries, Public Health Foundation of India, Gurugram, India; b Sangath, New Delhi, India; c Sapien Labs, Arlington, TX, USA; d Department of Global Health and Social Medicine, Harvard Medical School and the Harvard Chan School of Public Health, Boston, MA, USA

**Keywords:** Serious game, LMIC, cognition, neurodevelopment, early childhood development, mobile health

## Abstract

**Background**: Assessment of cognitive development is essential to identify children with faltering developmental attainment and monitor the impact of interventions. A key barrier to achieving these goals is the lack of standardized, scalable tools to assess cognitive abilities.

**Objective**: This study aimed to develop a tablet-based gamified assessment of cognitive abilities of 3-year-old children which can be administered by non-specialist field workers.

**Methods**: Workshops among domain experts, literature search for established and gamified paradigms of cognitive assessments and rapid review of mobile games for 3-year-old children was done to conceptualize games for this study. Formative household visits (N = 20) informed the design and content of the games. A cross-sectional pilot study (N = 100) was done to assess feasibility of the tool and check if increasing levels of difficulty and the expected variability between children were evident in game metrics. In-depth interviews (N = 9) were conducted with mothers of participating children to assess its acceptability.

**Results**: Six cognitive domains were identified as being integral to learning – divided attention, response inhibition, reasoning, visual form perception and integration and memory. A narrative, musical soundtrack and positive reinforcement were incorporated into the tool to enhance participant engagement. Child performance determined level timers and difficulty levels in each game. Pilot data indicate that children differ in their performance profile on the tool as measured by the number of game levels played and their accuracy and completion time indicating that it might be possible to differentiate children based on these metrics. Qualitative data suggest high levels of acceptability of the tool amongst participants.

**Conclusions**: A DEvelopmental assessment on an E-Platform (DEEP) has been created comprising distinct games woven into a narrative, which assess six cognitive domains, and shows high levels of acceptability and generates metrics which may be used for validation against gold standard cognitive assessments.

## Background

The lives of hundreds of millions of children, in particular from socially disadvantaged populations, are blighted by the impact of adversities in early life on cognitive development. Unsurprisingly, low and middle-income countries (LMICs) bear the bulk of this burden with over 200 million children under the age of 5 years failing to reach their full development potential in early childhood [1,2]. South Asia, particularly India, contributes the highest number (approximately 65 million) to this vulnerable group due to the disproportionately high burden of risk factors, such as poverty, in this region [3]. This suboptimal development is reflected in the poor education attainment of Indian children where though most children are enrolled in primary school, a quarter of them have education outcomes which are significantly below their grade level [4,5].

A key obstacle in optimizing the developmental potential of children in LMICs like India is the lack of scalable, standardized tools that can assess and monitor a child’s cognitive ability before they enter school, facilitating the targeting of effective interventions [6]. Commonly used tools are proprietary and require skilled professional expertise to administer and hence are difficult to scale-up in population-based routine surveillance programs [7–10]. Additionally, the massive paucity of skilled child development professionals and large maldistribution of these scarce personnel to urban locations and the private sector, further enhances inequities [11,12]. This results in an inability to establish regular child developmental surveillance systems which can track early cognitive development in children and mitigate both the personal and societal costs of inaction [13]. Importantly, missing subtle cognitive delays during early childhood, when the brain is maximally plastic and can benefit from targeted interventions that have been demonstrated to be effective in improving child developmental outcomes, is a limitation to attainment of cognitive potential [14–16].

In order to overcome these barriers, low-cost, scalable tools for assessment of cognitive abilities that are amenable for delivery by non-specialized community-based workers, such as those who routinely assess child growth, are urgently needed [17,18]. Creating tools that are context independent would allow for their use in diverse settings to generate normative curves for cognitive development that can be used globally. The Infant and Young Child Development Package (IYCD) and International Guide for Monitoring Child Development (GMCD) are examples of scalable tools that have been developed to fill this gap [19–21]. However, such tools are dependent either on care-giver responses, which often differ based on the respondents awareness of child development, or assessor observations, which requires extensive and continuous training.

The increasing availability of low-cost tablet computers and ubiquitous coverage of smartphones in most countries, including India, provides a unique opportunity to directly assess child performance on cognitive assessment tests [22,23]. It has been demonstrated that children as young as 2 years of age can make simple meaningful gestures on tablet screens (for example, tap, drag and swipe) with 3-year-olds being able to perform more complicated gestures such as rotate, flick and drag and drop [24,25]. Numerous established cognitive assessment paradigms like the NIH toolbox, CANTAB and CNS Vital Signs have already been adapted for administration on tablets [26–28]. However, there is increasing evidence that gamification of assessments enhances participant engagement and thus, there has been a thrust to develop ‘serious games’ which aim at cognitive assessment in children [29].

Recently, assessments of symptoms of developmental disorders such as Autism Spectrum Disorders (ASD) and Attention Deficit Hyperactivity Disorder (ADHD) have been gamified [30,31]. There is also some indication that these games might be able to provide metrics that are potentially sensitive to measuring change brought about through interventions [32]. Given the emerging evidence of effectiveness of gamified interventions for these disorders [33–37], the utility of such platforms can potentially extend from assessment of symptoms to delivery of targeted interventions and subsequent measurements of improvements in outcomes.

Amongst games for typically developing children, the focus has been largely on assessment and optimization of educational outcomes such as mathematical and reading skills and have thus targeted older school-aged children [38–41]. This approach misses out on the younger years, when children’s brains are maximally plastic and responsive to interventions [14,15]. The literature around gamified assessment of underlying cognitive domains which are integral to all forms of learning and which can be used with younger preschool children is limited though promising. A few games have been developed to measure discrete cognitive domains such as executive function, attention and memory and these have been tested on a wider age-range. Metrics such as game accuracy and completion time have been shown to differ according to difficulty of the task and age of participants [42]. The potential utility of these gamified tablet-based tasks for cognitive assessment in young children is further supported through preliminary evidence of their reliability and validity against gold standard assessments of child development [43,44].

The importance of assessing cognitive abilities in young children as a measure of their readiness for school lies in the fact that children identified to be lagging behind can be referred for early interventions thereby reducing the adverse impact on their academic performance upon entry in school [45]. We thus aimed to develop a tablet-based game called DEEP (DEvelopmental assessment on an E-Platform), which translates to light in Indian languages, and assesses a range of domains of cognition in preschool children. This paper describes the formative work and pilot study which have addressed the following questions – (1) which domains of cognition should be assessed in 3-year-old children and what games are available to assess them, (2) what are the optimal environmental conditions for delivery of a tablet-based gamified assessment of cognition in rural India, (3) what are the design elements that are most engaging for 3-year-old children and conducive to administration by non-specialist field workers and (4) does the gamified cognitive assessment have the potential to differentiate children based on their performance (5) what is the feasibility of the use of a gamified cognitive assessment tool and what is its acceptability to participants.

## Methods

### Study site and participants

The sample for this cross-sectional study consisted of 120 children (61 girls and 59 boys, numbers in each study phase are below) aged 34–40 months in 123 villages in Rewari district of the state of Haryana in North India. Rewari district is predominantly agricultural (70% rural households) with some industrial sectors [46]. These children were randomly sampled from databases maintained by village sub-centres and the project team through prior work in the community. Families with a 3-year old child were approached by a team comprising two non-specialist graduates (assessors) recruited from the local community and informed about the project. If they expressed interest in participating, written consent of the parent/primary care-giver was taken and an appointment was scheduled as per their convenience. The demographic data of the study sample is summarized in Table 1.

**Table 1.** Demographic and prior smartphone exposure details of participants in the three sets of household visits that were conducted in this study.

Demographic details	Formative visit 1 (N = 10)	Formative visit 2 (N = 10)	Pre-pilot visit (N = 100)
	% or Mean (SD)		
Mean age, months	37.4 (1.6)	37.1 (2.3)	37.2 (1.0)
Gender, male	50%	40%	50%
Mean height, cm	88.8 (4.8)	91.4 (3.3)	90.2 (3.9)
Stunted (HAZ< – 2SD)	50%	11%	31.3%
Mean weight, kg	11.9 (1.7)	11.5 (1.1)	11.8 (1.4)
Underweight (WAZ< – 2SD)	44%	22%	29%
Mean head circumference, cm	–	48.7 (1.3)	47.6 (1.9)
Exposure to schooling			
Attending private preschool	50%	30%	11%
Attending Anganwadi Centre	70%	10%	12%
Prior smartphone exposure			
Previously used smartphones	40%	80%	86%
Frequent user (within last 24 hrs)	–	62.5%	62%
Reasons for using smartphone			
Listening to music	–	70%	57%
Watching videos	–	20%	29%
Playing games	–	70%	59%
Seeing photos	–	20%	23%
Browsing internet	–	0%	2%

### Procedures

This study was conducted in three phases described below and summarized in Table 2.

**Table 2.** Summary of the iterative process of game development.

Phase	Approach	Games	Learnings	Impact on game development
Phase 1 – Game conceptualization	Expert consultation and rapid review of cognitive and gaming literature	N/A	Identified memory, reasoning, response inhibition, divided attention, visual form perception and visual integration as domains of cognition integral to learning Numerous games testing individual cognitive domains are available off-the-shelf and have a wide range of front-end graphics, music and user interfaces	Identified off-the-shelf games that primarily assess each of these domains. Multiple games testing the same cognitive domain were identified to enable characterization of front end features that are attractive/distracting to children
Phase 2 – Formative visits	Household visits stage 1 (N = 10)	Off-the-shelf games targeting the domains identified during conceptualization	Children were easily engaged by tablet-based games for 20–30 minutes Tapping on tablets came naturally, drag-and-drop is a tougher gesture Identified elements of game interface that were conducive or distracting for gameplay Identified the importance of use of narrative, music and positive reinforcement	Designed game to be 20–30 minutes long Administered tapping games first followed by drag-and-drop games Used elements of game design that were conducive for game play Integrated an upbeat musical soundtrack and confetti/applause as positive feedback; brainstormed potential narratives that would engage children
	Household visits stage 2 (N = 10)	Alpha versions of game being developed in this study	Some games showed saturation of child performance while others were too difficult Children enjoyed the preliminary narrative tested	Number and order of difficulty levels in each game and their timers determined Independent games integrated into a first-person narrative
Phase 3 – Pilot study	Household visits (N = 100)	Beta version of game being developed in this study	Number of children playing game levels decreases as difficulty increases Accuracy decreases as difficulty increases Completion time increases as difficulty increases	DEvelopmental Assessment on an E-Platform (DEEP) ready for validation against a gold standard measure of development

#### Phase 1 – game conceptualization

The aim of this phase was to conceptualize a game that would have the potential to assess cognitive abilities in preschool children that are integral to being successful in a learning environment. An international team comprising one developmental paediatrician, one psychiatrist, one clinical psychologist, two neuroscientists, two machine learning experts and a group of game developers conducted consensus workshops to conceptualize the game developed in this study. A rapid review of the literature of established and gamified paradigms of cognitive assessment and off-the-shelf tablet-based games for 3-year-old children was also done by the team.

#### Phase 2 – formative visits

Two sets of 10 household visits each constituted the formative work for this study. A team comprising two assessors and one senior researcher spent approximately one hour in each household; the initial 15 minutes were devoted to a short conversation with the mother/caregiver that probed the child’s daily routine. Parents also responded about whether or not their child attended any government (Anganwadi) or private preschool and if they had ever used a smart device (Table 1). The assessors then engaged the child with tablet-based games for up to 30 minutes which the child played using their finger, and the last 15 minutes were used to get the mother/caregiver’s opinion on the visit and for assessors to record their feedback on environmental aspects of the session including the number of family members present during the visit and the presence of disruptions. All conversations with the family, including delivery of game instructions to the child, were conducted in their preferred language (Hindi/Haryanvi). The role of the accompanying senior researcher was to take detailed observations of the assessment session which were tabulated using Excel and analysed through discussions amongst authors.

##### Household visits stage 1

The first set of 10 household visits were conducted from September to October 2017 and were used to inform the optimal household conditions for delivery of gamified assessments on tablet computers in rural settings, which included an analysis of environment, family and child factors. A Microsoft Surface Pro 4 tablet containing a suite of off-the-shelf non-modifiable previously developed games for 3-year-old children was taken into the household [47–49]. We ensured that these games comprised a variety of user interfaces to enable characterization of front-end features that are conducive or distracting for gameplay among children with no prior exposure to tablets and observations were made as to how children engaged with such games. These observations were used to optimize the content and design of the front-end of the tool developed in this study. Keeping scalability in mind, we decided to develop our game for an Android operating system and thus all further testing was done on considerably less expensive Samsung tablets.

##### Household visits stage 2

A game design agency (Quicksand Design Studio Pvt Ltd) developed initial versions of the game specifically for testing (alpha versions) using age-appropriate mechanics and design elements informed by the first set of formative visits. A second set of 10 children were administered these games on a Samsung tablet in household visits conducted between January-February 2018 and their performance recorded by the backend data was iteratively used to develop the content and design for a revised version of the game ready for testing (beta version). Feedback from the assessors was also incorporated into design of the beta version.

#### Phase 3 – pilot study

The beta version of the game was tested on 100 children through household visits conducted between February-May 2018. A team of 2 assessors administered the game on a Samsung Galaxy Tab E tablet and the backend data was used to derive metrics which provided indicators of game performance. In all games, all taps (intentional or accidental) on correct objects were recorded as correct taps, incorrect objects as incorrect taps and anywhere else on the screen as background taps. For dragging games, drags to correct positions were recorded as correct drags and anywhere else on the screen as incorrect drags. These were used to derive the accuracy (percentage correct taps/drags) with which a game was played. The timestamp for each tap/drag was collected to inform metrics such as completion time (time of the last correct tap/drag). We assessed if increasing levels of difficulty of the games and the expected variability between children were evident in the game metrics. Descriptive statistics was used to summarize, tabulate and visualize the data using Microsoft Excel and R [50].

#### Phase 4 – qualitative data collection and analysis

In-depth interviews were conducted with mothers of 9 children that participated in the pilot study. Participants were purposively sampled ensuring maximum variation through representation of children that did and did not engage with the tablet-based assessment. The interviews were conducted in participant’s homes in Hindi, recorded with permission, transcribed verbatim, translated to English and reviewed by the interviewer for accuracy. Interviews consisted of open ended questions designed to probe the following topics: (1) understanding of assessment during consenting, (2) family’s anticipation of the visit, (3) children’s interest in DEEP and (4) reasons for lack of instant engagement by the child. Data was analysed on NVIVO using Thematic Framework Analysis approach [51].

## Results

### Game conceptualization

Six cognitive domains emerged as primarily being integral for learning, and thereby school readiness: (1) response inhibition, a form of executive control that allows for suppression of unrequired actions; (2) divided attention, the ability to attend to multiple tasks at one time; (3) visual form perception and (4) visual integration, recognition of visual elements of objects and their integration into a whole; (5) reasoning, the use of logic to make decisions; and (6) memory; storage of information [8,10,52–55]. Since development of fine motor skills and cognition are known to be highly correlated [56], and fine motor skills are integral to any form of engagement with tablet-based games, we also included games that could potentially test manual processing speed and manual coordination [43].

### Optimal conditions for administration of the gaming assessment in rural Indian households

The first stage of 10 formative visits explored the optimal household conditions for administering tablet-based assessments. It was found that families were generally very welcoming and reacted positively to the idea of gamified assessments. Most families were able to direct us to a room within the house which was optimally lit for easy viewing of the tablet screen and free of loud noises to minimise distractions for the child. While some families had smartphones, none had ever seen a tablet computer before and this novelty often led to high interest levels amongst other adults and children in the family and their being present during the assessment. There was no influence of the number of family members present on disruptions that occurred during an assessment and, in cases when disruptions did occur, they were due to other family members assisting the child in playing the games or other children wanting to play on the tablet themselves. Based on these observations, families were requested to ensure that only one adult (preferably the mother/primary care giver) accompanied the child during the assessment to ensure the child’s comfort and this adult was then instructed not to assist the child during the assessment.

### Factors impacting gameplay

Four children (out of 10) had previously interacted with smartphones. Interestingly, prior exposure to smartphones had no effect on the child’s interest in the tablet-based games or on the time taken to familiarize with the device which was instant in most cases (7/10). Mothers of the remaining three children suggested that stranger anxiety, drowsiness and ill health were the likely reasons for their disinterest in the assessment. However, once engaged with the tablet, it was easy to sustain interest of all children with gameplay for 20–30 minutes. Our cognitive assessment tool was thus designed to be administered within this timeframe. In terms of response gestures, we found that tapping on the tablet came intuitively to the majority of children (9/10) while drag and drop was a more difficult gesture for children without prior smartphone exposure and required some practice to master. In order to prevent the need for children to switch between these gestures repeatedly, we decided to cluster our games according to the type of response required with tapping games being administered first followed by drag and drop ones.

Observations were made about elements of the user interface of these off-the-shelf games which were distracting or conducive to gameplay and these informed the design of the alpha version of our tool. For instance, it was found that complex, textured images confused children so simple objects in bold colours were chosen for our tool. A variety of user interfaces for games like matching shapes and jigsaw were tested and these informed the placement of objects on the screen. The use of music and positive reinforcement to maintain children’s engagement and optimize their performance on the game was recommended by caregivers and also observed by the researcher during these visits. Thus, an upbeat musical soundtrack was incorporated into our game and confetti appeared on the screen along with the sound of applause at the successful completion of every game level. Our game designer collaborators proposed that using a story to weave together discrete games was a promising way to keep children engaged and motivate them to complete the entire assessment and this was incorporated into the beta version.

### Testing alpha versions of the games to optimize acceptability to children and assessors

A second set of 10 household visits were conducted in which children played alpha versions of the games developed for this study to optimize their acceptability and ease of administration through an iterative process in which various front-end design features such as colours and placement of control buttons were modified. Eight of the 10 children visited had previously used smartphones. The backend data collected during these visits was used to determine the number and order of presentation of difficulty levels within games. For example, alpha versions of Hidden Objects (HO) and Jigsaw (JIG) contained only four difficulty levels but saturation of child performance was observed such that the fourth level was not tougher than the third and thus additional levels were added in the beta version. Similarly, alpha versions of Location Recall (LR) contained only three potential hiding places for the moon but this was gradually increased to five across game levels in the beta version.

Based on assessor feedback, in order to minimize decision making by them, a time limit was added for each level of a game. Backend data on children’s average completion time on the alpha version were used to determine optimal level timers. This was done to ensure that children unable to play a particular game level do not disengage from the assessment due to boredom or frustration. For instance, every level in HO timed out at 45 seconds while in Matching Shapes (MS), easier levels have 1-minute timers and this was increased to 2 minutes each for more difficult levels.

### Design of beta version of the game

**Figure 1.**
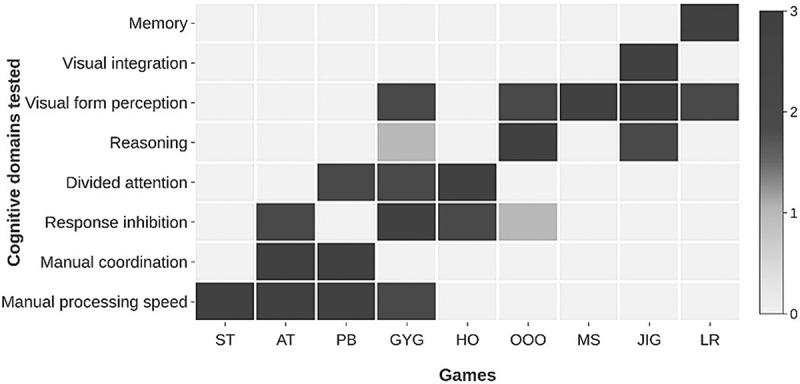
Cognitive domains tested by each game.

The learnings of the formative work (Table 2) were incorporated into the design of the beta version of the games developed for this study. While we have included games in this tool that primarily tap into specific domains of cognition, we expected that most games would tap into multiple cognitive domains and that multiple domains are represented in all the games (Figure 1). A summary of the final version of the nine distinct games in the tool is provided in Table 3. Given the evidence that the use of narratives in games increased participant engagement [57], these games were woven together into a story: while playing with its friends (different planets), the moon falls from the sky onto a park bench where a child is seated. Seeing the moon unhappy at being separated from its friends, the child embarks on a journey to take the moon back to them. The child and moon take off in a rocket but whenever they land on a planet, the moon hides from the child. In order to find the moon and continue the journey, the child has to first play a game (ST-JIG). Upon completion of the entire set of games, an animation shows the moon pleased to be reunited with its friends and the child returns home. A preview ‘demonstration’ mode was presented before the start of every new game in which the assessor administered standardized game instructions (see Table 3) verbally. To ensure that comprehension of language was not a limiting factor in the child’s ability to understand the instructions, on this screen the assessor would also first show the child how to play the game, and then assist the child till they are able to play independently. Assessors were given rules which had to be met while the child was playing independently to demonstrate their understanding of the game before they could proceed to the assessment mode (see Table 3). The demonstration screen allowed 5 attempts after which it was recorded that the child was unable to play that particular game. During the play mode, assessors only provided general encouragement to the child and did not repeat game instructions or assist the child in any manner. If not completed within its time limit, the game terminated and the child was allowed to continue on the ‘journey’ by playing the remaining games.

**Table 3.** The games used in the assessment.

Game name (abbreviation) and description	Snapshot	Primary domain tested	Instruction for play	Start rules	Difficulty levels (no. of levels)	Backend data metrics
Single Tap (ST) Single balloon presented which released stars when tapped.		Manual processing speed	Pop this balloon as fast as you can	Child pops the balloon 5 times	NA	Correct and incorrect taps with time stamps
Alternate Tap (AT) Two balloons presented. Each faded into a shadow when tapped while the alternate balloon retained its colour.		Manual processing speed and coordination	Pop these balloons alternately as fast as you can	Child pops the balloons alternately 3 times	NA	Correct, incorrect and background taps with time stamps
Popping Bubbles (PB) Balloons spawned from bottom to top.		Manual processing speed and coordination	Pop as many balloons as you can	Child pops 5 balloons	Increased speed of spawning balloons (2)	Correct and background taps with time stamps
Grow Your Garden (GYG) Water buckets and bugs presented randomly which, when tapped, resulted in a flower appearing and disappearing respectively.		Response inhibition	Touch the bucket, do not tough the bug	Child touches 3 buckets and avoids 2 bugs	Stimuli presentation changed from distinct to overlapping and time reduced from 3–1 second (4)	Correct, incorrect and background taps with time stamps
Hidden Objects (HO) Multiple monsters simultaneously hide in different locations		Divided attention	Touch the hiding place of the characters	Child taps correct locations and no incorrect ones	Increased number of characters with ratio of potential hiding places fixed at 1:2 (5)	Correct, incorrect and background taps with time stamps
Odd One Out (OOO) Four objects presented amongst which three were identical and one distinct.		Reasoning	Touch the object which is different from the other 3	Child taps correct object and no incorrect one	Objects differed on either colour, size, shape or category (4)	Correct, incorrect and background taps with time stamps
Matching Shapes (MS) Objects presented on the left with their outlines on the right. Each object had to be moved to its outline.		Visual form perception	Drag the objects to their matching shadows	Child is able to match 2 objects to their shadow	Increased similarity between objects presented together (5)	Correct and incorrect drags with time stamps
Jigsaw (JIG) Animal parts presented on the left with the outline of the complete animal on the right. Each part had to be moved to its outline to create the whole animal.		Visual integration	Drag the parts of the animal to its shadow to make a whole	Child is able to complete a 2-piece jigsaw	Increased number of pieces of jigsaw and similarity between them (6)	Correct and incorrect drags with time stamps
Location recall (LR) The moon hid behind one of multiple hiding places on a planet. The child had to recall the moon’s hiding location after completing each game listed above.		Memory	Touch the hiding place of the moon	Child taps correct location and no incorrect one	Increased number of possible hiding places (8)	Correct, incorrect and background taps with time stamps

### Feasibility and performance evaluation of the beta version of the game

**Figure 2.**
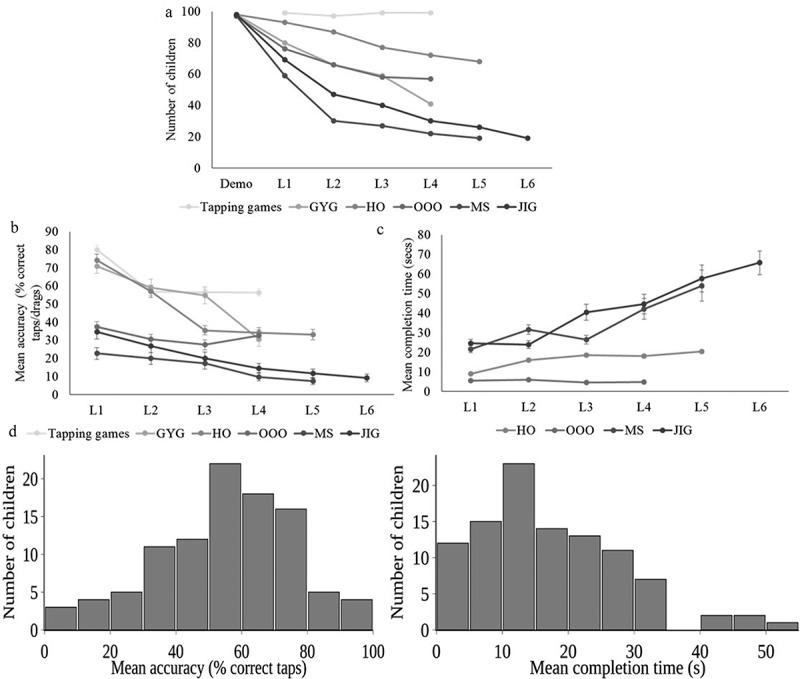
Performance of children on beta version the games during pilot study (n = 100); L1-L6 represent increasing difficulty levels within each game. (a) Number of children reaching game levels for all games. LR is not represented since it was integrated within the narrative and thus all children played all levels; (b) Mean accuracy as percentage correct taps/drags across game levels (error bars are standard error means); (c) Mean completion time for HO, OOO, MS and JIG (error bars are standard error means). Data for ST, AT, PB and GYG is not represented (see results for reasons). (d) Histograms of accuracy and completion time in LR.

This game was pilot tested on 100 children (104 families were approached and 4 refused consent either due to expectation of money, being against any governmental or non-governmental services or inability to spare time for the visit) and data metrics derived from the backend (Table 3) were used as indicators of game performance. A majority of these children (86%) had previously used smartphones. 92 of the 100 children tested attempted the demo of every game. Disruptions to the assessment were reported by assessors in three instances wherein the child was either distracted or bored and once when the tablet malfunctioned. Interestingly, while most children attempted the demo of a game, the number able to reach higher game levels decreased with each level of difficulty in all the games as we had expected. This trend is not visible when Single Tap, Popping Bubbles levels 1 and 2 and Alternate Tap are represented as levels 1–4 respectively (see Table 3 and Figure 2(a)) since these have been administered independently and not as difficulty levels within a single game. MS appeared to be a particularly challenging game for this set of participants with just 30 children proceeding past level 1. Mean accuracy decreases with increasing difficulty in all games except Odd One Out (OOO), where levels represent varying concepts (colour, size, shape and category) rather than increasing difficulty. The extremely low accuracy in MS further highlights it as being difficult for this group of children (Figure 2(b)). Figure 2(c) shows that, as expected, the mean amount of time taken to complete a game level also increases with increasing difficulty in most games with the exception of OOO. OOO completion time also has minimal variability in data indicating that some game metrics might perform better than others in differentiating children. Completion time is not reported for ST, PB and AT since they did not have an end-point and for Grow Your Garden (GYG) since it depended on the order of presentation of the stimuli and not on child performance. An example of a game which showed good variability in both the metrics of accuracy and completion time is LR (Figure 2(d)).

Thus, children seem to differ on the basis of their performance profile on this tool, from the subset of games which they attempt to the level of difficulty that they are able to complete within a game and finally their accuracy and completion time within game levels indicating that it might be possible to differentiate children using a combination of such metrics.

### Acceptability of the assessment to participants

86 mothers of the 100 children assessed in the pilot study were asked whether they liked the tablet-based assessment and would recommend it to others and all responded positively. In-depth interviews (see Table 4 for themes and quotes) conducted with a subset of these mothers indicated that all the respondents knew from the information given to them during consenting that the project involved an assessment of their child’s cognitive abilities. Most appreciated the opportunity to know about their child’s developmental status and were eagerly anticipating the visit. Only one mother indicated experiencing anxiety that the assessment might find some weakness in her child. The interviews revealed that most children were excited by the gamified assessment, enjoyed playing it and in fact, talked about it with their parents after the assessment was over and looked forward to the possibility of playing the game again. Confirming our observations during the formative visits, mothers of the few children that were not instantly engaged by the tablet indicated that they might have been experiencing stranger anxiety upon initial interactions with the assessors.

**Table 4.** Qualitative interview results.

Category	Theme	Quote
Before the visit	Understanding of assessment during consenting	*‘They [assessors] will tell me about my child’s mental development and I will also find out how smart he [the child] is’* Mother of 912#06#008#NA#01
	Family’s anticipation of visit	*‘I got to know that you would come and that made me happy.’* Mother of 922#01#016#DX#01 *‘I felt kind of nervous as to how he will perform. I also wondered that if he won’t be able to do these things then what if they find a weakness in him.’* Mother of 922#01#091#BY#01
During the visit	Children’s interest in DEEP	*‘[Child’s name] was happy and she was clapping and she was telling me that when madam will come again I will play again and burst balloons.’* Mother of 916#05#233#KU#01 *‘Even after two days of them leaving, [child’s name] asked me when they [assessors] were going to come back and if they would come back with the tablet.’* Mother of 912#06#008#NA#01
	Reasons for lack of instant engagement	*‘When a stranger from outside comes to the house, then the child gets a little scared on seeing them’* Mother of 912#06#008#NA#01 *‘It so happened [lack of instant engagement] because she [child] saw you all [assessors] for the first time and children do get a little nervous on seeing strangers.’* Mother of 916#05#391#RA#01

## Discussion

To the best of our knowledge, this is the first published study conducted in a rural setting of an LMIC aimed at developing an engaging, age-appropriate digital gamified assessment of cognitive development in preschool children for administration in households by non-specialist providers. The protocol for data collection on gamified cognitive assessments has been optimized for the settings of this study, which are likely to be generalizable to other low-income settings. Families in the community were enthusiastic and cooperative about the concept and application of gamified assessment tools. Disruptions due to external factors such as presence of observers or ambient noise were minimal. We observed that eighty percent of the children who participated in our research had prior exposure to smartphones. Additionally, we did not observe any impact of prior exposure on children’s inclination to play on tablets and ability to tap, increasing the generalizability of our assessment method. Children with limited smartphone exposure did find the drag and drop gesture more challenging, however, they could be taught this during the course of our interaction. Most studies cite enhanced participant motivation and usability by the target group as the reasons for gamification of cognitive assessments [29]. In our study too, almost all the children we approached were easily engaged by gamified assessments for relatively long periods of time with minimal encouragement. Similar to previously reported data, we also observed that they can easily learn to perform simple gestures on a tablet, such as taps or drag and drops, in a meaningful manner at 3-years of age [24,25].

The DEvelopmental assessment on an E-Platform (DEEP), developed in this study has been designed in an iterative manner with child performance and assessor feedback during formative visits informing game characteristics including difficulty within game levels and the amount of time for which an attempt is allowed. The pilot data reported here indicates the games are performing as per expectation with fewer children completing higher game levels. Decreased accuracy and increased completion time also indicate that difficulty is increasing across game levels. The variability with which children play these games is indicative of their potential to differentiate between children based on their performance. High completion rates and overwhelmingly positive responses from mothers of the participating children in in-depth interviews suggests high acceptability and feasibility of DEEP.

While physical development of a child is monitored through internationally accepted height-for-age (HAZ) and weight-for-age (WAZ) metrics, there are no such normative curves available for development of cognitive abilities, even in high-income countries. The absence of normative curves makes it challenging to adopt scalable methods to identify children faltering in their development, who could benefit from early interventions, and to monitor the impact of interventions. The scarcity of scalable, validated tools for assessment of cognitive development contributes to this challenge of tracking a key component of childhood development. DEEP, the tool developed in this study, consists of multiple distinct games which have been integrated through the use of a narrative which is free of language and cultural references and instead uses universal elements such as the moon, planets and monsters. This ensures that its utility can be pilot tested in culturally diverse settings with minimal need for adaptation. Since it has been developed for mobile devices, it would have tremendous capacity to be taken to scale through administration across multiple devices, synchronization of data on cloud servers and use of computing processes to continually update developmental norms.

This modular tool structure is also conducive to inclusion or exclusion of games based on their requirement in a study. Each game can be independently modified to alter, add or remove difficulty levels to increase their suitability to the target population. Thus, while its current design and content is informed by the performance of children residing in a rural setting, it has the potential to be expanded and made more challenging when urban or older children are tested. The cognitive domains assessed by this tool are similar to those commonly assessed in older children which also renders it suitable for mapping of cognitive growth trajectories through longitudinal studies in the long term. Given the emerging utility of games for cognitive training, the same platform could potentially be used to identify children faltering in their developmental trajectory, provide targeted interventions and monitor any improvements that might result from them. Apart from the cognitive domains discussed in this study, we foresee the ability of such a tool to expand to incorporate other domains of development such as socio-emotional and receptive and expressive language through gamification of traditional assessment paradigms. The validity of this tool, against the Bayley’s Scale of Infant and Toddler Development (BSID – 3rd edition), a gold standard of structured developmental assessment, is now being conducted in a larger sample of 3-year-old children. Once validated, we expect the low-cost, scalable gamified cognitive assessment tool to be able to address the major challenge in global early child development research of assessment of cognitive development in preschool children.

Limitations of the study – while DEEP has been administered by non-specialists, we acknowledge that their qualifications are higher than most community health workers that are a part of existing health systems in India and most LMICs. The ability of these health workers to administer the tool would need to be tested before it can be deployed at scale. However, we feel that the standardization of instructions and training that has emerged as a result of this pilot exercise will enable such task-shifting.

## Conclusion

In order to address a key obstacle in ensuring that children attain their optimal developmental potential, a DEvelopmental assessment on an E-Platform (DEEP) has been created comprising distinct games woven into a narrative which currently assesses six cognitive domains. Results of a pilot study shows high levels of its acceptability and an ability to generate metrics which may be used for validation against gold standard cognitive assessments. Once validated, such a tool would have potential to be administered at scale and aid in the identification of children who are impaired in their cognitive development thereby facilitating their timely referral to appropriate interventions.

## Paper context

To ensure that all children attain their full developmental potential it is essential to be able to assess their developing cognitive abilities. A key obstacle to this is the lack of a low cost, portable cognitive assessment tool suitable for preschool children. This study reports the design, development, acceptability and feasibility of a gamified assessment of cognition in 3-year-old children along with potential metrics which may be used for validation against gold-standard cognitive assessments.
